# Association between Daily Living Walking Speed and Walking Speed in Laboratory Settings in Healthy Older Adults

**DOI:** 10.3390/ijerph17082707

**Published:** 2020-04-15

**Authors:** Hisashi Kawai, Shuichi Obuchi, Yutaka Watanabe, Hirohiko Hirano, Yoshinori Fujiwara, Kazushige Ihara, Hunkyung Kim, Yoshiyuki Kobayashi, Masaaki Mochimaru, Eiki Tsushima, Kozo Nakamura

**Affiliations:** 1 Tokyo Metropolitan Institute of Gerontology, 35-2 Sakae-cho, Itabashi-Ku, Tokyo 173-0015, Japan; obuchipc@tmig.or.jp (S.O.); ywata@den.hokudai.ac.jp (Y.W.); h-hiro@gd5.so-net.ne.jp (H.H.); fujiwayo@tmig.or.jp (Y.F.); kimhk@tmig.or.jp (H.K.); 2 Gerodontology, Department of Oral Health Science, Faculty of Dental Medicine, Hokkaido University, Kita13, Nishi7, Kita-Ku, Sapporo 060-8586, Japan; 3 Faculty of Medicine, Hirosaki University, 5 Zaifu-cho Hirosaki City, Aomori 036-8562, Japan; ihara@hirosaki-u.ac.jp (K.I.); pteiki@hirosaki-u.ac.jp (E.T.); 4 Human Augmentation Research Center, National Institute of Advanced Industrial Science and Technology, c/o Kashiwa II Campus, University of Tokyo, 6-2-3 Kashiwanoha, Kashiwa, Chiba 277-0882, Japan; kobayashi-yoshiyuki@aist.go.jp (Y.K.); m-mochimaru@aist.go.jp (M.M.); 5 Towa Hospital, 4-7-10 Towa, Adachi-Ku, Tokyo 120-0003, Japan; kozo-nakamura-62@jcom.home.ne.jp

**Keywords:** daily living, frailty, global positioning system, physical function, walking speed

## Abstract

Although there is evidence on the predictors of adverse health outcomes in older individuals, walking speed has typically been measured in laboratory settings (LWS); LWS may be distinct from individuals’ actual walking speed in their daily lives (DWS). We examined whether DWS differs from LWS among older adults, and its association with physical frailty. Participants were 90 community-dwelling older adults. A five-meter normal (LWS_nor_) and maximum (LWS_max_) walking speed was measured with a stopwatch. DWS was measured using a global positioning system-related smartphone application for one month during their daily lives. DWS_avr_, DWS_max_, and DWS_sd_ were defined as the average, maximum, and standard deviation of walking speed for one month. Participants’ mean DWS_avr_ and DWS_max_ were 1.28 m/s and 2.14 m/s, respectively, significantly slower than the mean LWS_nor_ (1.42 m/s) and LWS_max_ (2.24 m/s); the intraclass correlation coefficient between DWS and LWS were 0.188 to 0.341. DWS was significantly correlated with grip strength, one-legged stance, and LWS. The area under the receiver operating characteristic curve of DWS_sd_ concerning pre-frailty was largest among DWSs, at 0.615, while that of LWS_nor_ was 0.643. The findings suggest that DWS differs from LWS and is associated with physical function and pre-frailty.

## 1. Introduction

Walking speed is an important measurement for assessing physical function in older individuals and patients since their lower-leg muscular strength, balance, and other functional capacities may deteriorate. Specifically, walking speed decreases with advanced age because of shorter step length, which is due to deteriorated muscular strength, decreased range of motion [1], and longer double-stance duration due to declined balance function [2]. Some studies among patients reported that walking speed could be a prognostic factor for vascular events in patients with stroke [3], and that slower walking speed was associated with deterioration in physical function among patients with secondary hip osteoarthritis [4]. In addition, the importance of prevention and improvement of frailty—a clinical state in which there is an increase in an individual’s vulnerability by aging for developing increased dependency and mortality when exposed to a stressor [5]—has increased in recent years. Various studies have recognized walking speed as a diagnostic indicator for physical frailty [6,7,8]. Moreover, previous studies have reported that walking speed predicts disability regarding instrumental and basic activities of daily living [9,10,11,12], cognitive decline [13], institutionalization [14], and even survival rates [15,16]; therefore, walking speed is a reliable predictor of adverse health outcomes among community-dwelling older adults.

However, these studies measured walking speed using an artificial walkway in a laboratory setting, where participants can intentionally change their walking speed. Individuals may behave very differently in medical settings than in their home environment. For instance, “white-coat hypertension” refers to a patient’s increase in blood pressure, perhaps because of evaluation apprehension during checkups [17]. Thus, walking speed measured in a laboratory may also be quite distinct from an individual’s true walking speed in their daily lives.

In recent years, various instruments have enabled assessments of daily living walking speed (DWS) [18,19,20,21]. Although measuring DWS may be useful for assessing regular walking ability and early stage frailty in daily life, to the best of our knowledge (except for studies that suggested that DWS is slower than walking speed in the laboratory (LWS) [21], or those that investigated the correlation between DWSs expressed as peaks and percentiles and LWS [22]), little research has examined the association between DWS and LWS among community-dwelling older adults. Additionally, no research has examined whether DWS is associated with physical function and frailty, as well as LWS. Therefore, to determine whether DWS can be used to assess physical function and frailty as well as LWS, we examined these associations by using a smartphone application, which was deemed reliable and valid in our previous study [20], with a sample of community-dwelling older adults.

## 2. Methods

### 2.1. Participants

This cross-sectional study utilized a population-based sample of community-dwelling adults. In 2018, we recruited community-dwelling older individuals who participated in comprehensive health checkups as a part of the 2011 Otassya-Kenshin Cohort Follow-Up Survey in Itabashi Ward, Tokyo, Japan. In 2011, we identified 7162 residents aged 65–84 years from nine areas and sent invitations to 6699 of them, excluding institutionalized or overlapping participants from our previous surveys. Consequently, 913 residents attended the health checkups as a baseline survey, and follow-up surveys have been conducted each year by adding in a new cohort of 65-year-olds. Details of the checkups in this cohort are described in our previous studies [23,24,25]. Overall, 769 older individuals underwent checkups in 2018, and we recruited the DWS survey participants at the checkup site; consequently, 106 accepted and participated in the DWS survey. Others did not participate for reasons such as being unwilling to use a smartphone or choosing not to take part. Although there were no specific exclusion criteria for the DWS survey, the measurements of physical function that require maximum effort for the participant during the checkup, such as grip strength and maximum walking, were not obtained from the following participants: those with angina pectoris, myocardial infarction, severe diabetes (with hypoglycemic attack, a fasting plasma glucose level of 200 mg/dL or higher, or complications of retinopathy or nephropathy), or severe hypertension (systolic blood pressure of 180 mmHg or higher or diastolic blood pressure of 110 mmHg or higher), which were assessed through nurses’ interviews.

We asked participants to carry their smartphones and perform their daily activities as usual for one month. A software application to measure walking speed in daily life (see the next section for details) was installed on the participants’ smartphones. If a participant did not have a smartphone, a smartphone with the installed software was loaned to him/her. We assumed the target sample size, *n* = 85, which is the required sample size for determining the walking speed difference of 0.1 m/s and the correlation coefficient of 0.3 (significant based on power analyses using G*Power 3.1 [26]). The software was maintained and operated by InfoDeliver Co., Ltd. (Tokyo, Japan).

### 2.2. Measures

#### 2.2.1. DWS and LWS Measurements

DWS was measured using a smartphone with a global positioning system (GPS) application. The application used in this study employed a system that we had evaluated for reliability and validity in our previous study [20]. We preliminarily confirmed that the application in this study was as accurate as the version reported in the previous study.

The application measured participants’ walking trajectory using position information acquired from the smartphone’s GPS. When a stable walking trajectory is detected for over 20 m, the walking speed is continually measured until there is an interruption in the walking. Once the application starts, walking speed is measured in the background while the participant walks outdoors (since the GPS is limited to outdoor use) during daily life; the measurements are performed passively with no additional action required by participants and without participants’ conscious awareness. While there were differences owing to participants’ frequency of going outside and lifestyle variables, approximately 50 to 1000 walking speed measurements were recorded per participant during the one-month period. In this study, DWS_avr_ was defined as the average value of the walking speeds measured for more than 50 walking bouts for one month during participants’ daily lives. We confirmed that walking speeds measured by this method during daily life were normally distributed, and the average value could be used as a representative value in daily life [20]. The means (standard deviations (SDs)) of skewness and kurtosis for the walking speed distributions in the current study were 0.481 (0.381) and 0.597 (1.090), respectively; therefore, these near-zero values indicate the data were normally distributed. In addition, DWS_max_ and DWS_sd_ were calculated from the maximum value and standard deviation of the walking speeds measured for one month during participants’ daily lives.

A stopwatch was used to measure LWS_nor_ and LWS_max_ while the participants walked at a normal and maximum pace during the checkups. Participants walked 5 m through a walkway, with the 3 m at the beginning and end of the walkway being used as acceleration and deceleration phases; walking speed within the intermediate area was calculated to denote LWSs [24,25]. LWS_nor_ was measured once. LWS_max_ was measured twice, and the better of the two results was used.

#### 2.2.2. Other Measurements

Body composition and physical performance were also measured during the checkups. Appendicular skeletal muscle mass (ASM) was estimated with bioelectrical impedance analysis using the InBody 720 device (InBody Inc., Seoul, Korea). The skeletal muscle index (SMI) was calculated as follows: ASM (kg)/height (m^2^). The grip strength of the dominant hand was measured using a Smedley-type dynamometer (AS ONE Corporation, Osaka, Japan). Participants performed one trial. For one-legged stance, the participants were instructed to stand with their arms at their side and with both legs together on the floor, and then keep one leg away from the floor and continue standing as long as possible. The leg that was elevated was the choice of each participant. The time from the point when they elevate their leg to the point when they lose their balance was measured, and its upper limit level was defined as 60 s. Participants performed two trials, and the better time was used. The presence or absence of chronic disease, such as hypertension, stroke, heart disease, diabetes, and osteoarthritis, were also assessed through nurses’ interviews during the checkups.

#### 2.2.3. Physical Frailty Assessment

To assess physical frailty, we used the five criteria from the Japanese version of the Cardiovascular Health Study [27]: weight loss, weakness, slowness, exhaustion, and low activity. Participants who did not have any of these components were considered robust, those with one or two components were considered pre-frail, and those with three or more were considered frail. 

The Kihon Checklist (KCL) is a valid 25-item questionnaire for predicting frailty and dependency among community-dwelling older adults [28,29]. “Weight loss” corresponded to participants who answered “yes” to question number 11: “Have you lost more than 2–3 kg in the last six months?”. “Weakness” corresponded to cases in which participants’ grip strength was under 26 kg for men or under 18 kg for women. “Slowness” corresponded to cases in which participants’ LWS_nor_ was less than 1.0 m/s. “Exhaustion” corresponded to cases in which the participant answered “yes” to question number 25: “In the last two weeks, I have felt tired for no particular reason”. “Low activity” applied to cases in which participants did not engage in light exercise or physical activity on a regular basis. In addition, total KCL score was assessed.

### 2.3. Statistical Analyses

Descriptive statistics were first calculated. The differences in the characteristics between the DWS-analyzed participants and the other participants were examined with t-tests or chi-square tests. The differences between the mean DWS_avr_ and LWSs, and DWS_max_ and LWSs were examined with paired t-tests. The intraclass correlation coefficients (3,1) between DWS_avr_ and LWSs, and DWS_max_ and LWSs were calculated, and an F-test with a true value of 0.6 was conducted to assess associations between LWSs and DWSs. Pearson’s correlation coefficients between DWSs, LWSs, the other measurements, and the KCL score were also examined. Since this study aimed to examine whether DWS could be used to assess physical function and frailty as well as LWS, a comparison of DWS with LWS was necessary, and age and sex adjustments were not performed.

The associations between each walking speed and the frailty group were investigated using receiver operating characteristic (ROC) curve analyses. Additionally, cut-off value, sensitivity, and specificity were calculated based on the Youden index. The accuracy of the ROC analysis was evaluated using areas under the curve (AUC) along with a chi-square test. SPSS 25.0 J (IBM Japan, Ltd., Tokyo, Japan) and STATA 15.0 (StataCorp LLC, College Station, Texas, USA) were the software packages used for the analyses.

### 2.4. Ethical Considerations

This study was conducted in accordance with the 1964 Declaration of Helsinki and its later amendments. All participants provided written informed consent to participate, and the study was approved by the ethics committees of the Tokyo Metropolitan Geriatric Hospital and Institute of Gerontology (no. K120; 2018).

## 3. Results

Fourteen participants whose DWS was not recorded more than 50 times, and two participants who did not have both LWS measurements were excluded from the analyses. Consequently, 90 participants (34 men and 56 women, aged 65–83 years) were eligible for the full analyses. The mean age was 71.8 (SD = 5.59) years. The prevalence of robust, pre-frailty, and frailty was 68.9%, 31.1%, and 0.0%, respectively (Table 1). DWS-analyzed participants were significantly younger, had longer one-legged stance times, had lower KCL scores, and had faster DWS and LWS than the other participants. Consequently, DWS-analyzed participants included significantly more robust people than the other participants. There were no significant differences between the DWS-analyzed participants and the other participants with regards to body height, body weight, SMI, grip strength, and prevalence of main chronic diseases.

The mean DWS_avr_ and DWS_max_ were 1.28 m/s and 2.14 m/s, respectively, which were significantly slower than the mean LWS_nor_ of 1.42 m/s and LWS_max_ of 2.24 m/s (Table 2). The intraclass correlation coefficient between DWSs and LWSs were 0.188 to 0.341, and the F-test results were non-significant (Table 2).

DWS_avr_ was significantly correlated with age, SMI, grip strength, one-legged stance, and LWSs. DWS_max_ was significantly correlated with age and LWSs. DWS_sd_ was significantly correlated with age, grip strength, and LWSs. LWS_nor_ was significantly correlated with the KCL score and LWS_max_. LWS_max_ was significantly correlated with SMI, grip strength, the KCL score, and LWS_nor_ (Table 3).

**Figure 1:**
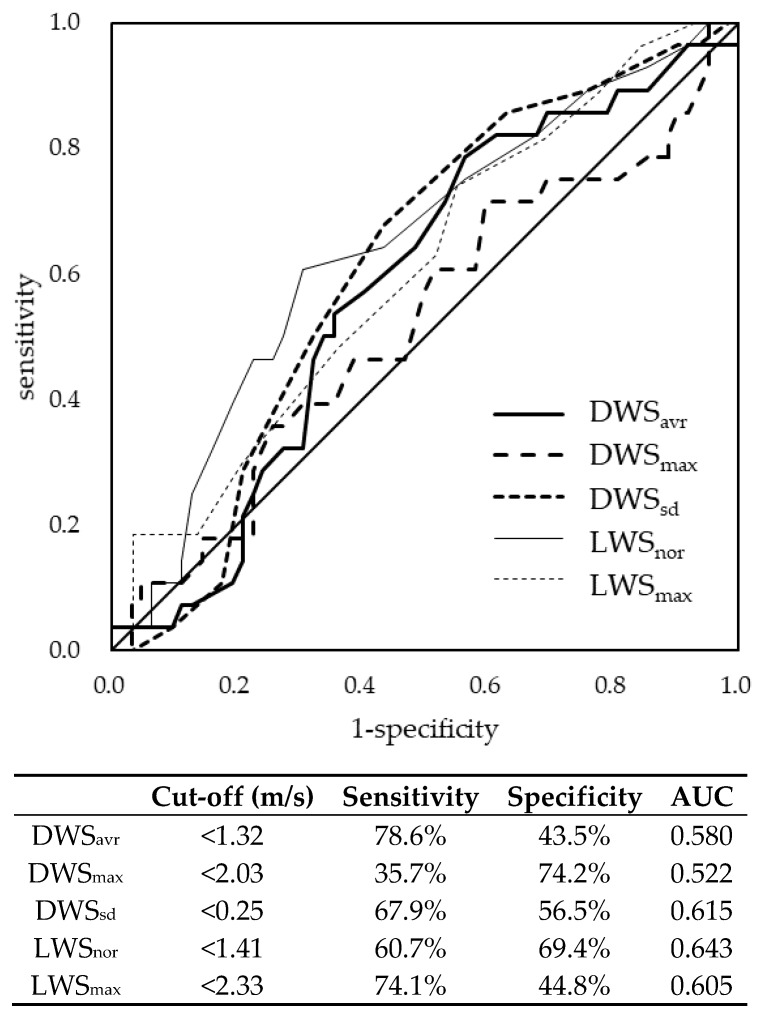
Receiver operating characteristic curve of daily living walking speed and walking speed in the laboratory regarding pre-frailty. DWS: daily living walking speed, LWS: walking speed in the laboratory, avr: average, max: maximum, sd: standard deviation, nor: normal, AUC: area under the curve.

The cut-off values for distinguishing between robust and pre-frailty based on the ROC curves were <1.32 m/s for DWS_avr_, <2.03 m/s for DWS_max_, and <0.25 m/s for DWS_sd_. They were <1.41 m/s for LWS_nor_ and <2.33 m/s for LWS_max_. The AUC for DWS_avr_, DWS_max_, DWSsd, LWS_nor_, and LWS_max_ regarding pre-frailty were 0.580, 0.522, 0.615, 0.643, and 0.605, respectively (Figure 1). The difference between the those AUCs was non-significant (*p* = 0.495).

## 4. Discussion

This study examined whether DWS differs from LWS and their associations with physical function and frailty among community-dwelling older adults. We revealed that DWS_avr_ and DWS_max_ differed from LWS_nor_ and LWS_max_, respectively, and that DWSs were associated with physical function and physical frailty, similar to LWSs.

Since a smartphone application was used to measure DWS, we examined the representativeness of our study sample. Compared with a previous study measuring LWSs in a large-scale population [30], the ratio of participants with a history of chronic disease was lower, and participants’ physical functioning was higher in the current study. LWSs in the current study were faster than those in the previous study (1.29 m/s and 1.94 m/s, respectively); therefore, the current study may have included healthier participants than in the previous studies. Moreover, no participants in the current study were categorized as frail, and the prevalence of pre-frailty was lower than in a prior study investigating community-dwelling older Japanese adults [31]. Thus, healthy participants who could use smartphones and who did not meet the criteria for frailty may have been included in this study. Indeed, DWS-analyzed participants had higher physical functioning than those who were not analyzed. However, there were no significant differences in body size nor the prevalence of main chronic diseases between the two groups; thus, the results should be interpreted as a tendency in healthy older adults with consideration to these characteristics.

The present results revealed that the participants’ DWS was slower than their LWS. In the only prior study that examined the difference between DWS and LWS [21], although DWS was measured by an acceleration sensor and was slower than reported in the current study (1.15 m/s in people aged 60–69 years), DWS was still slower than LWS (1.22 m/s in people aged 60–69 years), as in the current study. Previous research indicated that walking speed reflects not only individuals’ walking circumference, but also their mood [32]. Therefore, DWS could reflect factors other than walking ability. In addition, DWS could be measured over longer distances while LWS was only measured over 5 m. Since a previous study reported that short-distance walking speed may overestimate long-distance walking capacity [33], DWS—which included more long-distance walking—may be slower than LWS.

However, as DWSs were correlated with SMI and physical performance measures, DWS likely reflects the participants’ physical function. LWS was also correlated with those physical parameters similar to a previous study [34]. Since moderate correlations were observed between DWSs and LWSs, it could be argued that DWS reflects the participants’ physical function, similar to LWS. Additionally, LWSs were significantly correlated with the KCL score. Conversely, DWSs did not show a significant correlation with the KCL score. The KCL contains a comprehensive domain of frailty [35]. In other words, LWS may be associated more with comprehensive frailty, which includes physical, oral, cognitive, and social components.

The ROC analyses also revealed that the AUC for LWS_nor_, in comparison to DWSs, may be more associated with pre-frailty. However, there was no significant difference between DWSs and LWSs. Since the AUC of DWS_sd_ concerning pre-frailty was the largest among the DWSs (close to the value of LWSs), DWS can also be utilized to screen for pre-frailty, similar to LWSs. Given that LWS is an established measurement associated with many adverse health outcomes, DWS could be utilized to assess these related outcomes. Since DWS can be measured over the long-term and in different situations compared to LWS, using it to assess various adverse health outcomes would be beneficial in the future.

### Study Limitations

One study limitation was that the study participants were not randomly selected, and therefore two-thirds of the participants were considered robust, with no participants in the frailty category. As a result, our data can only address the relationship between walking speed and pre-frailty. This is attributed to the DWS measurement using a smartphone application. Indeed, we found that DWS-analyzed participants were significantly younger and had higher functional capacity than the other participants. In the future, verification of the results of this study is needed in samples containing frail participants, as well as a detailed examination of the association between DWS and the components of physical frailty. Moreover, walking speed may depend on age and sex; however, this study was not designed to analyze age and sex subgroups and did not adjust for these. In the future, these subgroup analyses should be conducted after increasing the sample size and power.

Additionally, DWS may include a variety of contexts for daily walking, such as catching a bus, going shopping, walking for exercise, or walking with a companion. Thus, the effects of walking context should be investigated. We did not examine the participants’ cognitive and social functioning and how such variables could be related to walking speed and physical frailty. Since this study employed a cross-sectional design, the association between DWSs and future frailty has not been evident. Dissemination and continued use of DWS as a measurement tool will hopefully expand research into these domains.

## 5. Conclusions

This study revealed that DWS, when measured by a smartphone GPS, differed from LWS. In addition, DWS was associated with physical function and pre-frailty, as was LWS. In the future, DWS could be used to assess individuals’ physical function and frailty risk in their daily lives.

## Figures and Tables

**Table 1:** Participants’ characteristics and differences between daily living walking speed and walking speed in the laboratory.

Variables	DWS-Analyzed Participants (*n* = 90)			Other Participants (*n* = 679)			*p* ^†^
	Sample with Complete Data, *n*	Mean	SD	Sample with Complete Data, *n*	Mean	SD	
Age (years)	90	71.8	5.59	679	73.8	6.81	0.003
Height (cm)	90	158.2	9.28	679	156.7	9.20	0.147
Weight (kg)	90	57.5	11.58	679	56.3	11.28	0.340
SMI (kg/m^2^)	88	6.56	1.01	675	6.48	1.06	0.465
Grip strength (kg)	86	27.8	8.19	657	27.5	8.64	0.714
One-legged stance (s)	90	56.7	11.65	673	46.1	21.07	<0.001
KCL score	83	3.0	2.97	585	4.2	3.47	0.002
DWS_avr_ (m/s)	90	1.28	0.115	-	-	-	-
DWS_max_ (m/s)	90	2.14	0.189	-	-	-	-
DWS_sd_ (m/s)	90	0.24	0.028	-	-	-	-
LWS_nor_ (m/s)	90	1.42	0.216	672	1.35	0.273	0.007
LWS_max_ (m/s)	85	2.24	0.387	643	2.08	0.457	0.001
		*n*	%		*n*	%	
Female	90	56	62.2	679	409	60.2	0.717
Hypertension	90	37	41.1	676	287	42.5	0.808
Stroke	90	4	4.4	676	47	7.0	0.370
Heart disease	90	13	14.4	676	116	17.2	0.518
Diabetes	90	7	7.8	676	83	12.3	0.213
Osteoarthritis	90	21	23.3	676	88	13.0	0.009
Frailty (J-CHS)	90			679			<0.001
Robust		62	68.9		320	47.1	
Pre-frailty		28	31.1		327	48.2	
Frailty		0	0.0		32	4.7	

^†^*t*-test or chi-square test, SD: standard deviation, DWS: daily living walking speed, LWS: walking speed in the laboratory, avr: average, max: maximum, nor: normal, J-CHS: Japanese version of the Cardiovascular Health Study.

**Table 2:** Differences between DWSs and LWSs.

	*n*	DWSs (m/s)		LWSs (m/s)		*p* ^†^	ICC (95% CI)	*p* ^‡^
		Mean	SD	Mean	SD			
DWS_avr_ vs. LWS_nor_	90	1.28	0.115	1.42	0.216	<0.001	0.341 (0.145–0.511)	0.999
DWS_avr_ vs. LWS_max_	85	1.28	0.116	2.24	0.387	<0.001	0.226 (0.015–0.418)	1.000
DWS_max_ vs. LWS_nor_	90	2.14	0.189	1.42	0.216	<0.001	0.303 (0.103–0.479)	1.000
DWS_max_ vs. LWS_max_	85	2.14	0.192	2.24	0.387	0.016	0.188 (−0.025–0.385)	1.000

^†^ Paired *t*-test, ^‡^ ICC (3,1), F-test with true value 0.6. SD: standard deviation, DWS: daily living walking speed, LWS: walking speed in the laboratory, avr: average, max: maximum, nor: normal, ICC: intraclass correlation coefficient, CI: confidence interval.

**Table 3:** Pearson’s correlation coefficients between daily living walking speed, walking speed in the laboratory, age, appendicular skeletal muscle index, physical functions, and the Kihon Checklist score among participants.

	Age	SMI	Grip Strength	One-Legged Stance	KCL Score	LWS_nor_	LWS_max_
DWS_avr_	−0.348 **	0.233 *	0.408 **	0.233 *	−0.085	0.411 **	0.412 **
DWS_max_	−0.222 *	−0.065	0.068	0.048	−0.166	0.306 **	0.237 *
DWS_sd_	−0.208 *	0.128	0.244 *	0.121	−0.147	0.411 **	0.340 **
LWS_nor_	−0.145	0.067	0.085	0.161	−0.288 **	1	0.564 **
LWS_max_	−0.163	0.279 *	0.363 **	0.159	−0.257 *	0.564 **	1

* p < 0.05, ** p < 0.01. DWS: daily living walking speed, LWS: walking speed in the laboratory, avr: average, max: maximum, SD: standard deviation, nor: normal, SMI: appendicular skeletal muscle index, KCL: Kihon Checklist.
